# Relation of Malnutrition and Nosocomical Infections in Cancer Patients in Hospital: An Observational Study

**DOI:** 10.1155/2022/5232480

**Published:** 2022-08-16

**Authors:** Bianca Tabita Muresan, Martín Núñez‐Abad, Ana Artero, Jaime Rios Rios, Alberto Jacobo Cunquero-Tomás, Vega Iranzo, Javier Garrido, Ana Jiménez-Portilla, Carlos Camps Herrero, Carlos J. Sánchez Juan

**Affiliations:** ^1^Servicio de Endocrinología y Nutrición, Hospital General Universitario de Valencia, Valencia, Spain; ^2^Fundación Hospital General Universitario de Valencia, Valencia, Spain; ^3^Servicio de Oncología Médica, Hospital General Universitario de Valencia, Valencia, Spain; ^4^Departamento de Medicina, Universidad de Valencia, Valencia, Spain; ^5^Servicio de Urgencias Hospitalarias, Hospital Clínico Universitario de Valencia, Valencia, Spain; ^6^Centro de Investigación Biomédica en Red Cáncer CIBERONC, Madrid 28029, Spain; ^7^Molecular Oncology Laboratory, Fundación Investigación, Hospital General Universitario de Valencia, Valencia, Spain; ^8^Unidad Mixta TRIAL, Centro Investigación Príncipe Felipe-Fundación Investigación, Hospital General Universitario de Valencia, Valencia, Spain; ^9^Department of Medicine, Universitat de València, Valencia, Spain

## Abstract

**Aim:**

To investigate the relation between malnutrition and nosocomial infections (NI) in hospitalized cancer patients.

**Methods:**

This observational, cross-sectional, noninterventional, descriptive study was conducted in a 500-bed university hospital in Valencia (Spain). Adult cancer patients admitted to the oncology ward were consecutively enrolled regardless of their nutritional status between November 2019 and March 2020. Patients were nutritionally assessed 24 to 48 hours after admission. Body weight, height and BMI, body composition through measurement of bioelectrical impedance analysis (BIA), and muscle strength and functionality using hand grip strength (HGS) were prospectively collected. The diagnosis of malnutrition and sarcopenia was assessed using the Global Leadership Initiative on Malnutrition (GLIM) criteria and the European Working Group on Sarcopenia in Older People (EWGSOP) criteria, respectively. Patients were followed up during their hospital stay or outpatient oncology visits to identify possible NI.

**Results:**

A total of 107 patients were included in this study (mean age 66 years; 66.4% were men). The most frequent reason for admission was cancer treatment (19.6%), followed by infections (18.7%) and digestive tract symptoms (18.7%). Overall, 77.5% (83/107) of the patients were malnourished at admission according to the GLIM criteria, while 52.3% (56/107) were sarcopenic. Nosocomial infections (NI) were significantly more frequent in malnourished (52.1%; 25/48) and severely malnourished (42.1%; 8/19) patients, compared with well-nourished patients without malnutrition (25%; 10/40; *p*=0.035). The mean length of hospital stay was 13.9 days, significantly longer in patients with an NI compared to those without infections (18.6 vs. 10.8 days, *p* < 0.024).

**Conclusion:**

This study evidenced the need to implement a routine protocol for the nutritional assessment and support of cancer patients at risk of malnutrition and sarcopenia to reduce the risk of NI during their hospital stay.

## 1. Introduction

Cancer patients have a high risk of malnutrition due to metabolic derangements related to the neoplastic process and insufficient nutrient intake due to loss of appetite and frequent oncology treatments with adverse effects [1, 2]. The prevalence of malnutrition in cancer patients ranges from 20% to 70% depending on the patient age, cancer type, and cancer stage [1]. Malnutrition is more prevalent in older patients and those with gastrointestinal tract, head and neck (H&N) and lung cancers [1].

Among hospitalized cancer patients, malnutrition tends to worsen during hospital stays [3, 4]. It has been associated with a greater risk of complications [5], longer hospital stays [6], poorer tolerance and response to treatments [7], shorter survival [8, 9], and a significant decline in health-related quality of life [6, 10].

Sarcopenia is a progressive decline in muscle strength due to loss of skeletal muscle mass and dynapenia is an age-related loss of strength and muscle function [7, 11]. Both conditions are frequently reported in cancer patients, are associated with a higher risk of tumor recurrence and decreased survival, and lead to greater disability, immobilization, and risk of infections that may imply higher rates of hospitalization [7, 11–16].

Nutrition and infection interact through different pathways, the most important being an impairment of nonspecific and cell-mediated immunity [17, 18]. Nosocomial infections (NI) are one of the many consequences of malnutrition in a hospital setting, affecting care and outcomes in cancer and increasing healthcare costs [19]. Cancer patients are more vulnerable to developing severe infections (especially NI) owing to the malignancy itself and its treatments [20, 21]. Earlier studies have shown that 2.1% to 48% of cancer patients develop NI, varying widely across regions [22–26]. A study of critically ill cancer patients admitted to intensive unit care found that 40.7% of the patients contracted NI, with a rate of 4.6/100 patients-days [27].

Limited data are available on the relationship between nutritional status and other nutritional parameters and the risk of NI in hospitalized cancer patients. This study aimed primarily to establish the relationships that may exist between malnutrition according to the Global Leadership Initiative on Malnutrition (GLIM) criteria and NI in cancer patients admitted to hospital. Additionally, the study sought to define how sarcopenia may determine the occurrence of NI and how NI may affect the length of hospital stay. The prevalence of malnutrition, sarcopenia, and dynapenia at hospital admission by type of cancer were exploratory objectives.

## 2. Materials and Methods

### 2.1. Study Design and Patient Population

This observational, cross-sectional, noninterventional, descriptive study was conducted in a 500-bed university hospital in Valencia (Spain). From November 2019–March 2020, all adults (≥18 years) diagnosed with a solid tumor who were admitted to the oncology ward and gave their written consent were eligible and consecutively enrolled in the study. The nutritional status of the patients was recorded prospectively; however, data related to infection and type of cancer were collected retrospectively. Patients were enrolled regardless of undercurrent disease, cause of admission, age, or nutritional status. Patients were excluded if they had anasarca, orthopedic prostheses, extremity amputation, or pacemakers. Patients with a life expectancy <72 hours were also excluded.

The following demographic and clinical characteristics at admission were recorded: age, the reason for admission, intensive care unit stay, tumor location, tumor stage (localized tumor, locally advanced tumor, and advanced/metastatic tumor), performance status measured by Eastern Cooperative Oncology Group (ECOG), type of treatment, and line of treatment.

### 2.2. Study Assessments

Patients were assessed 24 to 48 hours after admission by the same trained dietician. Patients were followed up during their hospital stay. To identify NI happening after discharge from the hospital, they were followed up on outpatient oncology visits or during hospital readmission as usual clinical practice.

Endocrinologists and oncologists followed a nutritional assessment protocol to collect body weight, height and BMI, body composition through bioelectrical impedance analysis (BIA) measurement, and muscle strength and functionality by hand grip strength (HGS) at hospital admission. In addition, the diagnosis of malnutrition and sarcopenia were assessed using the GLIM criteria and European Working Group on Sarcopenia in Older People (EWGSOP) criteria, respectively.

Weight was measured with a 0.1 kg adjustable weighing scale (Omron® BF511, Omron Corporation, Japan). Height was calculated with a stadiometer (Seca®, model 220, Seca Ltd., United Kingdom) ranging from 60–200 cm. The BMI was calculated as weight divided by height squared (kg/m^2^). As part of anamnesis, the previous body weight was collected at 3 different times (3 months, 6 months, and 1 year before the date of admission).

BIA measurements were performed with a single-frequency instrument (BIA-101 BIVA®; Akern S. r. l, Italy). The device utilizes a root mean square current of 0.25 mA at a constant frequency of 50 kHz. Two electrodes were positioned on the dorsal surfaces of hands proximately to the metacarpal-phalangeal and 2 electrodes on the dorsal surfaces of feet proximately to metatarsal-phalangeal joint and with a greater distance of 4-5 cm between them. The patient was lying supine on a bed with legs apart and arms not touching the torso. Manufacturer-supplied equations (Bodygram PLUS®; Akern S. r. l, Italy) were used for calculating body composition. BIA measurements included resistance (Rz), reactance (Xc), phase angle (PA), fat-free mass (FFM, kg), fat-free mass index (FFMI, kg/m^2^), and fat mass (FM, kg). PA is the ratio between the 2 electrical measurements of Rz (electrical resistance of the tissue to the passage of current) and Xc (reactance capacity of cell membranes). PA represents an indicator of the general condition of the body having a normal value from 4–9. FM includes all the lipids that can be extracted from adipose tissue and other body tissues, as well as cutaneous and visceral fat. FFM includes muscle, bone, minerals, and other nonfat tissues, containing approximately 73% water, 20% protein, and 7% minerals. FFMI is the amount of fat-free mass about their height which is obtained by dividing FFM by the squared height of the patient.

HGS was quantified with a Jamar hydraulic dynamometer 5030J1 (Sammons Preston Rolyan, Chicago, United States). All patients were comfortably seated in a chair with their arm close to the trunk. The shoulder of the arm holding the dynamometer was in an adduction position, the elbow flexed to 90°, and the forearm and wrist were in a neutral position. Three maximal consecutive measurements were performed on each hand, with 60 seconds of rest between them to avoid muscle fatigue. The mean value of these measurements was calculated.

### 2.3. Diagnosis of Malnutrition, Sarcopenia, Dynapenia, and Nosocomial Infections

**Figure 1:**
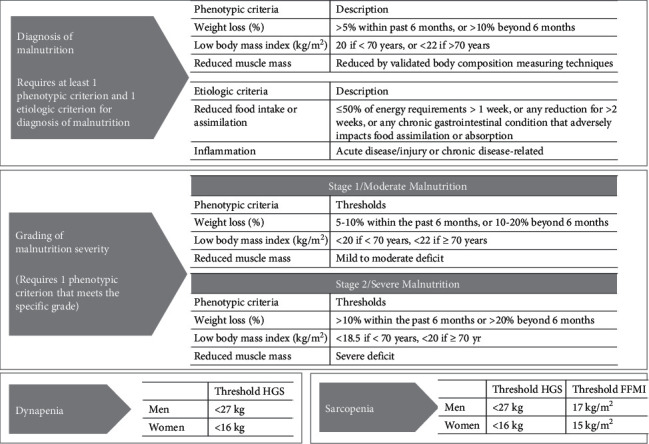
Summary of the criteria followed for the diagnosis of malnutrition, dynapenia, and sarcopenia in the study population [12, 28]^*∗*^. HGS: hand grip strength. ^*∗*^For further details, lectors are invited to review the consensus reports.

The diagnosis of malnutrition was made following the phenotypic and etiologic criteria established by the GLIM group. The phenotypic assessment included: nonvolitional weight loss and/or low BMI (<70 years old or reduced muscle mass), while the etiological evaluation included reduced food intake or assimilation and inflammatory condition. The severity of malnutrition was defined as moderate or severe, based on the thresholds of the phenotypic criteria [28] (Figure 1).

The diagnosis of sarcopenia was established according to the definition of the European Working Group on Sarcopenia in Older People (EWGSOP-2) based on the parameters of muscle mass and muscle strength [12] (Figure 1).

Dynapenia was diagnosed based on the cut-off points defined by the EWGSOP-2, using the highest HGS value when the mean values of each hand were compared (Figure 1).

NI was defined as all infections acquired from the hospital after 48 hours of admission or occurred 3 days after hospital discharge or within 30 days of any intervention, regardless of location and infectious agent. The techniques to evaluate and confirm NI were very varied, from blood cultures, urine cultures, and stool cultures to imaging tests such as X-rays, computerized tomography, and even surgical techniques such as exploratory laparoscopy.

### 2.4. Statistical Analysis

Stata 14 statistical program was used to perform descriptive analyzes. Means, medians, and proportions were calculated for quantitative and qualitative variables. Distribution normality was analyzed using Shapiro–Wilk tests. A Pearson correlation coefficient was calculated to measure the association between malnutrition (by measuring weight, BMI, FFMI, and PA) and the occurrence of NI, sarcopenia and the occurrence of NI, and between NI and the length of hospital stay. *p* values <0.05 were considered statistically significant.

### 2.5. Ethical Considerations

This study was approved by the Ethics Committee of Hospital General Universitario of Valencia on 7 February 2020. Patients gave their written consent before study entry.

## 3. Results

### 3.1. Patients' Characteristics

A total of 107 patients were assessed in this study. The most frequent reason for admission was cancer treatment (19.6%) followed by infections (18.7%) and digestive tract symptoms (18.7%). The mean age was 66 (range 33–95) years; 66.4% were men. The most common tumor sites were the lungs (26.2%), colorectal (13.1%), and the gastroesophageal tract (12.1%). The majority (79.4%) had an ECOG performance status ≥1. Overall, 70.1% of the patients had advanced disease and 74.8% were in active oncology treatment: 38.3% received first-line therapy, while 36.4% received second or subsequent lines; 24.3% were on treatment for symptoms control only (Table 1).

### 3.2. Anthropometric and Nutritional Status Assessments

**Figure 2:**
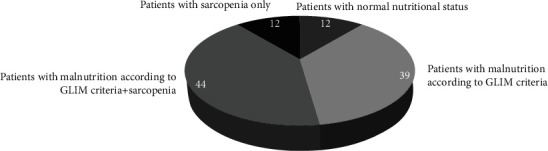
Distribution of study population by nutritional status (malnutrition, sarcopenia, and normal status).

Anthropometric and body composition measurements at admission are shown in Table 2. Overall, 77.5% (83/107) of the patients were malnourished at admission according to the GLIM criteria, and 52.3% (56/107) were sarcopenic. Of these, 41.2% (44/107) of the patients were diagnosed with malnutrition and sarcopenia. Only 11.2% of the patients (12/107) had a normal nutritional status (Figure 2).

### 3.3. Prevalence of Malnutrition, Sarcopenia, and Dynapenia by the Type of Tumor

The prevalence of moderate and severe malnutrition, dynapenia, and sarcopenia by tumor type in hospitalized cancer patients is shown in Table 3.

Patients with sarcoma (33%), lung cancer (25.0%), and gastroesophageal cancer (23.1%) had moderate malnutrition, while different proportions of patients with pancreatic-biliary cancer (66.7%), lung cancer (50%), and colorectal cancer (50%) were severely malnourished. In addition, 77.8% of patients with H&N cancer were severely malnourished, 88.0% had dynapenia, and 66.7% had sarcopenia. 87% of patients with breast cancer and 67.3% with gastroesophageal cancer had dynapenia. Patients with sarcoma, pancreatic-biliary cancer, H&N cancer (33.3% each), and gastroesophageal cancer (30.8%) had decreased FM.

### 3.4. Malnutrition, Sarcopenia, and Nosocomial Infections

40.2% (43/107) of patients developed NI during admission, mostly respiratory tract infections (24.3%), followed by urinary tract infections (7.5%) and bacteremia (6.5%). 12 patients (11.2%) had more than 1 NI during the hospital stay (Table 4).

Weight, BMI, and FFMI were significantly lower in patients who acquired NI (*p* < 0.05 in all cases). PA was not associated with NI (*p*=0.569) (Table 5).

**Figure 3:**
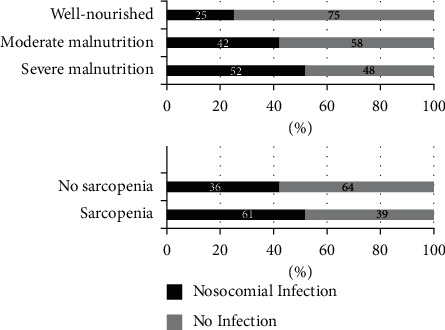
Percentage of nosocomial infection by a diagnosis of malnutrition or sarcopenia in cancer inpatients.

NI were significantly more frequent in patients with severe malnutrition (52.1%; 25/48) and moderate malnutrition (42.1%; 8/19), compared to well-nourished patients (25%; 10/40; *p*=0.035) (Figure 3(a)). NI was more common in patients with sarcopenia (61.1% vs. 36%, *p*=0.044) compared to that without sarcopenia (Figure 3(b)).

### 3.5. Length of Hospital Stay and Nosocomial Infection

The mean length of hospital stay was 13.9 days. The mean length of hospital stay was significantly longer in patients with a NI compared with those without infections (18.6 vs. 10.8 days, *p* < 0.024). There was no correlation between length of stay in hospital and nutritional status (*p*=0.477).

## 4. Discussion

This study reported the proportion of cancer patients with malnutrition, according to GLIM criteria and NI admitted to hospital with the primary aim of establishing the relation between malnutrition and NI in this group of patients. Our study showed that most of the patients had altered nutritional status and that nearly half of them developed NI during their hospital stay.

The percentage of malnourished patients in our study concurs with previously reported data in cancer hospitalized patients and according to GLIM criteria [29]. Compared to other studies reporting NI in cancer patients, the frequency of NI in our study was higher [20]. These differences may be partly attributed to the demographic and clinical characteristics of the study population. In our study, most of the patients had advanced stages of the disease and were in active oncological treatment at the time of admission. In this line, a similar percentage of NI was reported in critically ill cancer patients admitted to the intensive care unit [27]. Moreover, our *research* indicates that 18.7% of the study patients were admitted to the hospital due to an infection. These patients could also develop a nosocomial infection during hospitalization, such as *Clostridium difficile* due to the antibiotic. Therefore, all developed NI were identified, regardless of the cause of the hospital admission.

Based on our results, there was a relationship between the nutritional status of the study population and NI. Compared to well-nourished patients, NI was more frequent in moderately and severely malnourished patients. To our knowledge, this is the first study to describe the relationship between malnutrition defined by GLIM and NI in adult cancer patients admitted to the hospital.

Earlier studies have described the effect of malnutrition on health outcomes, including postoperative infections following cancer surgery. In patients undergoing pancreatic resection, malnutrition was associated with increased postoperative complications, including infections (odds ratio (OR): 2.63; 95% confidence interval (CI), 1.96; 3.52; *p* < 0.001) [30]. Similarly, the risk of surgical complications was significantly higher in malnourished patients with H&N cancer (OR: 8.5; 63% with vs. 17% without; *p* < 0.001) [31].

Regarding the relation between anthropometric body composition parameters and NI, data are very limited. Our study showed that patients with NI were significantly lower in weight, BMI, and FFMI. Earlier studies reported that underweight colorectal and gastric cancer patients undergoing resection developed more postoperative complications, including infections than patients of normal weight [32, 33]. In noncancer patient populations, FFMI has been identified as an independent predictor for postoperative infections [34, 35].

In this study, NI was more common in patients with sarcopenia compared to those without sarcopenia. Similar results have been reported in patients with digestive cancer who underwent surgery. In a study of patients with locally advanced esophageal cancer who proceeded to surgical resection, postoperative pneumonia occurred in 44.8% and 27.3% of patients with and without sarcopenia, respectively (OR: 2.17; 95% CI: 1.11; 4.26, *p* < 0.01) [36]. Patients with sarcopenia had an infection more frequently than those without sarcopenia (23.1% vs. 12.6%, *p* < 0.036) after primary colorectal cancer resection. This relation between sarcopenia and infections was also confirmed in the subgroup analysis of patients older than 65 years [37]. In patients with digestive carcinoma who had received surgery, sarcopenia was associated with an increased risk of infections (risk ratio (RR): 2.23, *p*=0.09), and severe infections (RR: 2.96, *p*=0.04) [38]. Sarcopenia was also identified as an independent risk factor for postoperative complications in patients with H&N cancer (OR: 4.5; 56% vs. 22%; *p* < 0.015) [31].

The mean length of hospital stay in our study was 8 days longer in patients with a NI compared with those without infections. Similar results have been reported in various studies regardless of the medical condition of the population [39]. In a study of patients with brain tumors admitted to a neurosurgery department, the mean length of hospital stay for all patients was similar to our study (14.34 days). However, the average length of hospital stay for patients with NI was 25 more days than for patients without infections (*p* < 0.01) [40]. Unlike our study, 20% of NI were neurosurgical site infections which are associated with significant morbidity and a longer hospital stay and require complex treatment including long-term antibiotic therapy [41].

Although in our study, there was no correlation between length of stay in hospital and nutritional status, malnutrition defined by the GLIM criteria was significantly associated with length of the hospital stays (*P*=0.001) in a large multicenter retrospective analysis (*n* = 2,388) [29].

Our study also explored the nutritional status of patients at hospital admission by cancer type. Patients with H&N cancer presented the worse nutritional status, with most of the patients being malnourished and/or sarcopenic [1, 31]. In this group of patients, severe malnutrition is associated with the presence of dysphagia symptoms in 76% of the patients [42].

The current study has a series of limitations, and the findings need to be interpreted accordingly. First, there are inherent limitations to a descriptive, observational, single-center study in a small sample. This might lead to a center-specific bias, influencing data acquisition. Data related to infection and cancer type were retrospectively collected introducing potential bias due to the possibility of missing data. The study was conducted in a heterogeneous sample of cancer patients admitted to an oncology ward which limits the extrapolation of results to specific cancer populations. A single observer performed the nutritional measurements only once, assuming the correct calibration of measurement tools.

Despite the limitations mentioned above, the results of this study suggested that malnutrition may contribute to the occurrence of NI in cancer patients. These findings would encourage further research into nutritional assessment and interventions in order to improve the nutritional status of cancer patients admitted to a hospital and consequently prevent NI.

## 5. Conclusions

Overall, this study showed that a large percentage of cancer patients admitted to our ward were malnourished and/or sarcopenic. This group of patients contracted NI more frequently. Therefore, this study evidenced the need to implement a routine protocol for the nutritional assessment and support of cancer patients at risk of malnutrition and sarcopenia to reduce the risk of NI during their hospital stay.

## Figures and Tables

**Table 1:** Patients' characteristics at hospital admission (study population, baseline).

Characteristics	Value (*n* = 107)
Age (years), mean (range)	66.19 (34–95)

Gender, *n* (%)	
Male	71 (66.4%)
Female	36 (33.6%)

Tumor location, *n* (%)	
Lung	28 (26.1%)
Colorectal	14 (13.1%)
Gastroesophageal	13 (12.1%)
Head and neck	9 (8.4%)
Breast	9 (8.4%)
Urinary tract	8 (7.5%)
Gynecologic	8 (7.5%)
Sarcoma	6 (5.6%)
Pancreatic-biliary	6 (5.6%)
Others	6 (5.6%)

Tumor stage^*∗*^, *n* (%)	
Local	6 (5.6%)
Locally advanced	26 (24.3%)
Advanced	75 (70.1%)

Performance status, *n* (%)	
0	22 (20.6%)
1	40 (37.4%)
2	16 (14.9%)
3	19 (17.8%)
4	10 (9.3%)
Patients on active oncological treatment, *n* (%)	80 (74.8%)

Oncological treatment at admission, *n* (%)	
Chemotherapy	35 (32.7%)
Immunotherapy	15 (14.0%)
Chemotherapy + immunotherapy	10 (9.3%)
Target therapy	12 (11.2%)
Palliative care	15 (14.0%)
No previous treatment	12 (11.2%)
Others	8 (7.5%)

Therapy lines	
First-line	41 (38.3%)
Second-line	23 (21.5%)
Third or later lines	16 (14.9%)
Patients without active oncological treatment, *n* (%)	27 (25.2%)

Reason for hospital admission, *n* (%)	
Cancer diagnosis or treatment	21 (19.6%)
Infection or sepsis	20 (18.7%)
Digestive tract symptoms (vomiting, bleeding, jaundice, intestinal obstruction…)	20 (18.7%)
Pain	15 (14.0%)
Dyspnea symptoms (pulmonary thromboembolism, lung cancer progression, chronic obstructive pulmonary disease)	11 (10.3%)
Medication toxicity	10 (9.3%)
Others	10 (9.3%)

^*∗*^Tumor stages were defined as localized tumors: amall tumors which can be respected and are usually curable; locally advanced tumors: large but localized tumors and/or with regional lymph node involvement, but without distant involvement. Potentially curable and advanced tumors: metastatic tumors (distant tumor lesions). Usually not curable [28].

**Table 2:** Anthropometric and body composition measurements.

Measurements	Value (*n* = 107)
Anthropometric	
Weight (kg), mean ± SD	67.89 ± 14.68
BMI (kg/m^2^), mean ± SD	24.12 ± 5.6
Weight loss within the past 6 months (%), mean ± SD	6.83 ± 11
Hand grip strength (kg), mean ± SD	22.19 ± 10,3

*Body composition*	
FFMI (kg/m^2^), mean ± SD	18.65 ± 2.75
FM (kg), mean ± SD	15.60 ± 9.17
PA (º), mean ± SD	4.6 (±1.78)

BMI: body mass index; FFMI: fat-free mass index; FM: fat mass; PA; phase angle; SD: deviation standard.

**Table 3:** Prevalence of moderate malnutrition, severe malnutrition, dynapenia, and sarcopenia by type of tumor.

Type of cancer	*N*	Moderate malnutrition (%)^*∗*^	Severe malnutrition (%)^*∗*^	Dynapenia (%)^*∗*^	Sarcopenia (%)^*∗*^	Decreased FM (%)
Lung	28	25.0	50	61.7	7.1	14.3
Colorectal	14	7.1	50	74.7	28.6	28.6
Gastro-esophageal	13	23.1	46.2	67.3	30.8	30.8
Head and neck	9	11.1	77.8	88.0	66.7	33.3
Breast	9	0	22.2	87.0	0	11.1
Urinary	8	22.2	22.2	54.7	11.1	11.1
Gynecologic	8	0	33.3	65.7	0	0
Sarcoma	6	33.3	16.7	64.8	0	33.3
Pancreatic-biliary	6	0	66.7	66.7	16.7	33.3
Other tumors	6	42.8	26.6	57.1	0	28.6

FM: fat mass. ^*∗*^Percentage is calculated as per number of patients with nutritional status and a certain type of tumor divided by total number of patients in the study.

**Table 4:** Types and frequency of nosocomial infections during the hospital stay.

NI during hospital stay	% of patients^*∗*^
Patients with 1 NI during hospital stay	29.0%
Patients with >1 NI during hospital stay	11.2%

Types of nosocomial infections	
Respiratory tract infections/pneumonia	24.3%
Urinary tract infections	7.5%
Bacteremia	6.5%
Clostridium difficile-associated diarrhea	4.7%
Phlebitis	1.9%
Candidemia	1.9%
Abdominal cellulitis	1.9%
Aspergillosis infection	1.9%
Catheter infection	0.93%
SARS-CoV-2 infection	0.93%

NI: nosocomial infection; SARS-CoV-2: severe acute respiratory syndrome coronavirus 2. Percentage is calculated as per the number of patients with NI divided by the total number of patients in the study. Note: because some patients have more than 1 nosocomial infection, the total number of NI is higher than the number of patients with NI.

**Table 5:** Correlation of weight, BMI, FFMI, and PA with nosocomial infection in hospitalized cancer patients.

Parameter	Nosocomial infection	*N*	Mean	*p* value
Weight	No	64	70.62 kg	0.004
	Yes	43	63.67 kg	
BMI	No	64	25.5 kg/m^2^	0.002
	Yes	43	22.2 kg/m^2^	
FFMI	No	64	19.37 kg/m^2^	0.001
	Yes	43	17.65 kg/m^2^	
PA	No	64	4.59°	0.569
	Yes	43	4.64°	

BMI: Body mass index; FFMI: Fat free mass index; PA; phase angle.

## Data Availability

The datasets used and/or analyzed during the current study are available from the corresponding author on reasonable request.
